# “I Know that You Know that I Know”: Neural Substrates Associated with Social Cognition Deficits in DM1 Patients

**DOI:** 10.1371/journal.pone.0156901

**Published:** 2016-06-03

**Authors:** Laura Serra, Mara Cercignani, Michela Bruschini, Lisa Cipolotti, Matteo Mancini, Gabriella Silvestri, Antonio Petrucci, Elisabetta Bucci, Giovanni Antonini, Loretta Licchelli, Barbara Spanò, Manlio Giacanelli, Carlo Caltagirone, Giovanni Meola, Marco Bozzali

**Affiliations:** 1 Neuroimaging Laboratory, IRCCS Santa Lucia Foundation, Rome, Italy; 2 Brighton & Sussex Medical School, Clinical Imaging Sciences Centre, University of Sussex, Brighton, United Kingdom; 3 Department of Neuropsychology, National Hospital for Neurology and Neurosurgery, London, United Kingdom; 4 Department of Engineering University of Rome, “Roma Tre”, Rome, Italy; 5 Department of Geriatrics, Orthopaedic and Neuroscience, Institute of Neurology, Catholic University of Sacred Heart, Rome, Italy; 6 Neuromuscular and Neurological Rare Diseases Center, S. Camillo Forlanini Hospital, Rome, Italy; 7 Department of Neurology, Mental Health and Sensory Organs (NESMOS), Faculty of Medicine and Psychology "Sapienza" University, Rome, Italy; 8 Department of Clinical and Behavioural Neurology, IRCCS Santa Lucia Foundation, Rome Italy; 9 Department of Neuroscience, University of Rome ‘Tor Vergata’, Rome, Italy; 10 Department of Neurology, IRCCS Policlinico San Donato, University of Milan, Milan, Italy; University of Valencia, SPAIN

## Abstract

Myotonic dystrophy type-1 (DM1) is a genetic multi-systemic disorder involving several organs including the brain. Despite the heterogeneity of this condition, some patients with non-congenital DM1 can present with minimal cognitive impairment on formal testing but with severe difficulties in daily-living activities including social interactions. One explanation for this paradoxical mismatch can be found in patients’ dysfunctional social cognition, which can be assessed in the framework of the Theory of Mind (ToM). We hypothesize here that specific disease driven abnormalities in DM1 brains may result in ToM impairments. We recruited 20 DM1 patients who underwent the “Reading the Mind in the Eyes” and the ToM-story tests. These patients, together with 18 healthy controls, also underwent resting-state functional MRI. A composite Theory of Mind score was computed for all recruited patients and correlated with their brain functional connectivity. This analysis provided the patients’ “Theory of Mind-network”, which was compared, for its topological properties, with that of healthy controls. We found that DM1 patients showed deficits in both tests assessing ToM. These deficits were associated with specific patterns of abnormal connectivity between the left inferior temporal and fronto-cerebellar nodes in DM1 brains. The results confirm the previous suggestions of ToM dysfunctions in patients with DM1 and support the hypothesis that difficulties in social interactions and personal relationships are a direct consequence of brain abnormalities, and not a reaction symptom. This is relevant not only for a better pathophysiological comprehension of DM1, but also for non-pharmacological interventions to improve clinical aspects and impact on patients’ success in life.

## Introduction

Myotonic dystrophy type-1 (DM1) is the most common muscular dystrophy observed in adults [1]. It is caused by a CTG triplet repeat expansion within the myotonic dystrophy protein kinase (DMPK) gene located on chromosome 19q13.3, and whose inheritance is autosomal dominant [2]. DM1 is a multi-systemic disorder dominated by muscular impairment, but also involves other organs including the brain [1]. Previous literature has reported that most of the cognitive impairment observed in patients with non-congenital DM1 is driven by higher-order dysfunctions, such as planning, reasoning, monitoring, visuo-constructive abilities, etc., [1,3–8]. Indeed, when DM1 patients are formally tested on basic cognitive domains, such as memory, attention, and language abilities, they perform relatively well [5]. Structural [5, 9–10] and functional brain changes [4] were previously demonstrated in DM1 patients, and associated with both isolated cognitive deficits [5,9] and personality disorders [4]. However, a paradoxical mismatch exists between the widespread brain damage observed in DM1, the relative preservation of global cognition and highly impacting disabilities in daily activities. One possible explanation is that in non-congenital forms of DM1 this difficulty is due to a dysfunction of social cognition. Consistent with that, Kobayakawa and co-workers [11] have recently reported that patients with DM1 are impaired in tests assessing the theory of mind (ToM), an important component of social cognition functioning. ToM refers to the ability to infer other people’s mental states, thoughts and feelings. ToM is necessary to empathize and have a good relationship with others in social situations [12]. We hypothesize here that abnormal processing of social cognition, which is the putative core of DM1 higher-level dysfunctions and daily-living disabilities, has a specific neurobiological substrate and may be assessed in terms of brain connectomics within the framework of ToM. Previous task-related functional MRI (fMRI) studies demonstrated that ToM abilities depend on the integrity of several brain regions, mainly the medial prefrontal cortex, the cingulum, the precuneus, the temporal, and occipital regions (see [13] for a meta-analysis). It has also been reported that the neural basis underling the ToM is task-dependent [13]. Indeed, several tests can be used to assess ToM, some of them are story-based, which can be presented either verbally or visually, others are administered using non-story-based visual stimuli, such as the “Reading the Mind in the Eyes” test [14].

Against this background, it is therefore conceivable that one or more networks, rather than isolated regions, sub-serve ToM, and that abnormal connectivity within these networks might explain ToM deficits in DM1. Resting-state functional MRI (RS-fMRI) [15] is one of the most widely used methods to investigate brain connectivity in neurological and psychiatric diseases with the advantage of not requiring participants to perform any active task. RS-fMRI data can be analysed using different methodological approaches. One of them is based on the whole-brain analysis driven by graph theory [16–17], a mathematical approach that describes complex systems as networks [16–17]. In essence, the brain is represented by a number of regions (nodes) that are functionally connected to each other by the edges. Edges can be computed from RS-fMRI data. In this view, nodes with many connections are more critical (i.e., more “central”) for transferring information across the network, and are called “hubs”. Abnormal connectivity between “hubs” is believed to cause more relevant deficits than that between peripheral nodes [16–17].

To the best of our knowledge, no previous attempt was made to identify a “ToM” network using graph-theory approaches, or to assess the related functional connectivity changes in DM1. The present work was thus designed 1) to identify the so-called ToM-network in patients with DM1, and 2) to investigate the differences in the topological properties of the ToM-network between DM1 patients and healthy controls.

## Materials and Methods

### Participants

Twenty patients with a molecular diagnosis of DM1 were recruited from the Neuromuscular and Neurological Rare Diseases Centre at San Camillo Forlanini Hospital (Rome, Italy), from the Institute of Neurology at the Catholic University of Rome (Rome, Italy), and from the Department of Neurology, Mental Health and Sensory Organs (NESMOS), Faculty of Medicine and Psychology at the University of Rome “La Sapienza” (Rome, Italy). All recruited patients had a Mini Mental State Examination (MMSE) [18] score above 26 in order to exclude the presence of general cognitive impairment. Moreover, in order to exclude comprehension deficits in oral and written speech, two trained neuropsychologists (L.S. and M.B.) carefully assessed all DM1 patients based on a clinical interview. As detailed below, CTG expansion size within the DMPK gene was assessed for all DM1 participants and used to classify them according to the International Myotonic Dystrophy Consortium nomenclature [19]. The Muscular Impairment Rating Scale (MIRS) [20] was used to clinically characterise DM1patients. Eighteen healthy controls were recruited by distributing leaflets at the Santa Lucia Foundation (Rome) for MRI data collection only. Principal demographic characteristics of the participants are summarized in Table 1. Principal genetic and clinical characteristics of DM1 patients are summarized in Table 2. All participants were right handed as assessed by the Edinburgh Handedness Inventory [21]. All subjects underwent clinical assessment to exclude the presence of major systemic and neurological illnesses in controls, and pathologies different from known comorbidities in DM1 patients.

**Table 1:** Principal demographic characteristics of studied subjects.

	DM1 patients N = 20	HS N = 18	p-value
Mean (SD) age [years]	43.9(10.7)	42.7(12.4)	n.s. ^a^
Gender (F/M)	11.0/9.0	10.0/8.0	n.s. ^b^
Mean (SD) years of formal education	13.0(2.6)	15.0(3.2)	n.s. ^a^

^a^ t-test with 36 degree of freedom;

^b^ Chi-square

Abbreviations: DM1 = Myotonic dystrophy type 1; HS = healthy subjects.

**Table 2:** Principal genetic and clinical characteristics of patients with Myotonic dystrophy type-1.

	DM1 patients N = 20
**Age at onset:**	
Childhood-onset (age range: 6–16 years)	5(25.05)
Adulthood-onset (age range:18–60 years)	15(75.0%
**CTG triplet expansion** **(mean**± **SD)[range]**	462±260[150–1200]
**IDMC nomenclature:**	
E1 (CTG range: 50–150) (N and %)	1(5.0%)
E2 (CTG range: 151–500) (N and %)	12(60.0%)
E3 (CTG range: 501–1000) (N and %)	6(30.0%)
E4 (CTG range >1000) (N and %)	1(5.0%)
**MIRS stage:**	
Stage 1 (N and %)	0(0.0%)
Stage 2 (N and %)	6(30.0%)
Stage 3 (N and %)	11(55.5%)
Stage 4 (N and %)	**4(15.0%)**

Abbreviations: DM1 = Myotonic dystrophy type 1; IDMC = International Myotonic Dystrophy Consortium; MIRS = Muscular Impairment Rating Scale.

### Ethics statement

This study was conducted according to the principles expressed in the Declaration of Helsinki. The Ethics Committee of the Santa Lucia Foundation approved the study. Written informed consent was obtained from all recruited subjects before study initiation.

### Genetic assessment

Normal and proto-mutated alleles were analysed using “touch down” PCR on DNA obtained from peripheral blood leukocytes (PBL). Briefly, 50 pg of PBL-DNA were amplified in a 20μl volume with fluorescent labelled primer 101 and primer 102. Reactions were cycled through eight rounds at 94°C-30”, 68°C-30” (-1°C per cycle) and 72°C-30”, followed by 30 rounds at 94°C-30”, 60°C- and 72°C -30”. PCR products were then analysed using an Abi-Prism 310 Genetic Analyzer. Determination of expanded alleles was performed on 10 pg of PBL DNA, which underwent XL-PCR [22], and 1% agarose gel electrophoresis. PCR products were analysed by Southern blotting with subsequent hybridization to a ^32^P radiolabeled (CTG) 7-oligonucleotide probe and detected using autoradiography.

### General cognitive efficiency

As previously reported [5] all patients underwent Wechsler Adult Intelligence Scale-Revised (WAIS-R) [23–24] as measures of global cognitive efficiency.

### Theory of mind assessment

DM1 patients underwent ToM assessment using a non-story-based test, namely the “Reading the Mind in the Eyes” [14, 25] and a story-based test, namely the “Theory of Mind” [26].

The “Reading the Mind in the Eyes” test (RMET) [14, 25] allows an evaluation of social cognition, by assessing the ability to recognize the mental state of others based on the expressions around eyes only. DM1 patients were randomly presented with a series of 36 pictures of the eye regions (19 males and 17 females). Each picture was surrounded by four mental state descriptors represented by single words (e.g., friendly, cautious, bored, angry, etc.), with only one of them corresponding to the mental state expressed in the picture. Participants had to choose which one of the four descriptors best described what the person in the picture was “thinking or feeling”. In this test, the principal score is given by the number of descriptors correctly identified (hit-rates), the maximum score being 36. The hit-rates were transformed in z-scores using the Italian Normative data as a reference [25]. Patients with z-score below or equal to 1.5 were considered as impaired at RMET. We also computed the number of errors in males’ (EM) and females’ (EF) pictures separately.

The “Theory of Mind” (ToM-story) is a sub-test derived from the Social Cognition Battery [26]. This test is a modified Italian version of Happé’s stories [27] that requires subjects to read 13 short stories describing naturalistic social situations. Patients were asked to describe the characters’ motivation for their behaviour, with the subsequent ToM-story score indicating their comprehension of the situation. The maximum score was 13, and in accordance with the Italian Normative data [26] a score of ≤12 was classified as impaired.

The ToM assessment was available for 13 HS only (72.0%).

### Image acquisition and pre-processing of resting-state fMRI

All participants (patients and controls) underwent MRI at 3T, including a volumetric scan (3D Modified Driven Equilibrium Fourier Transform or MDEFT) and an RS-fMRI series collected during rest for 7 min and 20s resulting in a total of 220 volumes. During this acquisition participants were instructed to keep their eyes closed, not to think of anything in particular, and not to fall asleep. RS-fMRI images were pre-processed for resting-state fMRI using Statistical Parametric Mapping 8 (SPM8 http://www.fil.ion.ucl.ac.uk/spm/) and in-house Matlab scripts.

They underwent head motion correction (using the standard SPM8 realignment algorithm), compensation for slice-dependent time shifts and co-registration to the corresponding MDEFT. Each MDEFT-volume was segmented into white matter, grey matter and CSF maps using the standard SPM8 algorithm. The resulting grey matter images were used to compute each participant’s total grey matter volume. Segmentation derived normalization parameters were used to warp the motion and slice-time corrected RS-fMRI images into Montreal Neurological Institute (MNI) coordinates. In-house software was used to remove the global temporal drift using a 3rd order polynomial fit. Data were then filtered by regressing out movement vectors, and the average white matter and cerebrospinal fluid signal. The resulting images were then filtered using a phase-insensitive band-pass filter (pass band 0.01–0.08 Hz) to reduce effects of low frequency drift and high frequency physiological noise then smoothed with an 8 mm^3^ FWHM 3D Gaussian Kernel.

### Statistical analysis

Statistical analyses on behavioural and clinical data were performed using SPSS (SPSS Inc., Chicago, Illinois).

Demographic characteristics were compared between DM1 patients and HS using an independent sample T-Test for all variables with the exception of gender, which was compared using a Chi-square test.

Each score assessing the ToM was correlated, by using Pearson’s correlation coefficient, with demographic, clinical and genetic characteristics of DM1 patients. Moreover, to obtain a single comprehensive score of ToM (named ToM Composite score ToMCs, see Supporting information S1 File) to be used for correlation with MRI data, principal component analysis (PCA) was applied to the four available scores (i.e., RMET-hit rates, RMET-EM, RMET-EF, ToM-story). This composite score expresses a proxy measure of ToM abilities at individual level.

The correlations between ToMCs and network connectivity data were calculated using Network-based statistics [28], as detailed in the next section. For each considered global and local measure of connectivity (see below) a two-sample t-test was used to assess group differences between DM1 patients and controls in SPSS.

Finally, using a Chi-square test in SPSS, we performed a post-hoc analysis to compare between DM1 patients and HS the frequency of occurrence of a correlation between the main “hub” of the ToM network (emerging from graph theory analysis as described below) and all the other nodes in the network.

### Identification of the Theory of Mind network

In order to isolate a ToM network in patients with DM1, we used the “Networks-based statistics” (NBS) tool developed by Zalesky and co-authors [28]. This tool allows the correlation between any numerical score (such as the above described ToMCs) and the so-called “connectivity matrices”, representing the whole-brain network to be tested. The nodes, or the sub-networks, whose correlation with ToMCs was statistically significant were regarded as neuronal substrates for ToM abilities. In order to obtain a connectivity matrix for each participant, we first identified a set of 116 nodes defined by the automated anatomical labelling (AAL) atlas. Each node’s mean time course was calculated as the average of the fMRI time series from all voxels within a certain region. Correlation matrices were then obtained calculating the correlation between all pairs of nodes’ mean signals. In this way, we were able to represent inter-nodal connectivity by means of functional correlation between regional activities [28]. Correlation with ToMCs was based on one-sample t-test, using 5000 permutations and setting the significant p-value at 0.05 [corrected for multiple comparisons [28].

We assumed that the resulting network expresses the ToM-network, and therefore we set out to compare the topological properties of such a network between DM1 patients and controls. These properties, which characterise the network using special indices describing its shape and efficiency, can be estimated using the graph theory [16–17]. We used the Brain Connectivity Toolbox [17] and MATLAB custom-made scripts to explore global and local topological properties of the ToM-network in each participant’s brain.

For the purpose of the current study, we used those nodes that were significantly associated with the ToMCs by NBS analysis to construct the graph. Then, undirected binary connectivity matrices were built by thresholding the graphs (see the S2 file). The thresholds were determined in order to compare connectivity matrices with the same density values across all subjects, with density being the ratio between the total number of edges and the maximum possible number of edges within the network. Details of graph theory and its application to brain networks can be found elsewhere [16–17], together with a full description of all the indices that can be derived from it. Finally, we focussed on a subset of global properties, primarily assessing nodes’ segregation and integration. With respect to local metrics, we considered betweenness centrality, nodal degree, and nodal efficiency. Betweenness centrality is defined as the fraction of all the shortest paths passing through a given node; nodal degree expresses the number of connections for each node; and nodal efficiency is inversely correlated to each node’s paths length, and identifies the less efficient nodes along certain routes.

## Results

### Demographic, clinical and cognitive characteristics of studied subjects

Patients and controls did not differ significantly in mean age, gender or years of formal education (t = 0.29, Chi-square = 0.09, t = -1.97 respectively all p = n.s., see Table 1).

Table 2 summarizes genetic and clinical characteristics of all recruited patients. Most DM1 patients (15 out of 20, 75.0%) had an adulthood onset of disease, while 5 out of 20 patients (25.0%) had childhood-onset. The mean CTG triplet expansion was 462 ±260.6 ranging from 150 to 1200. Specifically, following the guidelines of the Myotonic Dystrophy Consortium (IDMC, 2000), one out of 20 patients (5.0%) was classified as E1 type, 12 out of 20 (60.0%) were classified as E2 type, 6 out of 20 (30.0%) were classified as E3 type, and 1 out of 20 (5.0%) were classified as E4 type. According to MIRS disease classification, 6 out of 20 (30.0%) were at the second disease stage, 11 out of 20 (55.5%) were at the third stage, 4 out of 20 (15.0%) were at the fourth stage.

As reported in Table 3, when considering the cognitive level the DM1 patients showed an intelligence quotient (I.Q.) in the range of normality: mean (SD) Total I.Q. = 108.5 (15.0); mean (SD) Verbal I.Q. = 102.8 (15.0); mean (SD) Performance I.Q. = 114.5 (15.2). Moreover, DM1 patients showed normal performances in each WAIS-R’s subtests, with the only exception being Vocabulary, which specifically assesses semantic knowledge. It is remarkable that the best performance was obtained by DM1 patients at the Comprehension subtest [mean (SD) score: 12(3.0)] indicating a normal level of comprehension of abstract social conventions, rules and verbal expressions.

**Table 3:** Performances obtained by DM1 at WAIS-R.

	Age-adjusted score
***Verbal subtests*:**	
**Information (mean 10; SD 3)**	10 (2.2)
**Digit span (mean 10; SD 3)**	10 (3.0)
**Vocabulary (mean 10; SD 3)**	7.5 (2.8)
**Arithmetic (mean 10; SD 3)**	9.8 (4.2)
**Comprehension (mean 10; SD 3)**	12 (3.0)
**Similarities (mean 10; SD 3)**	9.5 (4.5)
***Performance subtests*:**	
**Picture completion (mean 10; SD 3)**	10.7 (3.0)
**Picture Arrangement (mean 10; SD 3)**	10.3 (3.2)
**Block design (mean 10; SD 3)**	9.2 (2.3)
**Object Assembly (mean 10; SD 3)**	9.3 (2.8)
**Digit Symbol (mean 10; SD 3)**	10.6 (3.0)
***Intelligent quotient*:**	
**Total IQ (mean 100; SD 15)**	108.5 (15.0);
**Verbal IQ (mean 100; SD 15)**	102.8 (15.0);
**Performance IQ (mean 100; SD 15)**	114.5 (15.2)

### Theory of mind assessment

Sixteen out of 20 patients with DM1 (80% of the sample) reported pathological performances in either test assessing ToM abilities, predominantly at ToM-story (mean±SD Errors 3.3± 1.6). Specifically, when considering the number of stories failed at ToM-story test by DM1 patients, 2 out of 16 (12.5%) failed 1 story; 4 out of 16 (25.0%) failed 2 stories; 3 out of 16 (18.7%) failed 3 stories; 5 out of 16 (31.2%) failed 4 stories, 1 out of 16 (6.2%) failed 6 stories, and finally, 1 out of 16 (6.2%) failed 7 stories. Additionally, a negative correlation was found between patients’ RMET hit-rates scores and their CTG triplets’ expansion (r = -0.49, p = 0.04). No other significant correlations were found.

In the HS group we observed a ceiling effect in both measures assessing ToM.

### Resting-state fMRI analyses

**Fig 1:**
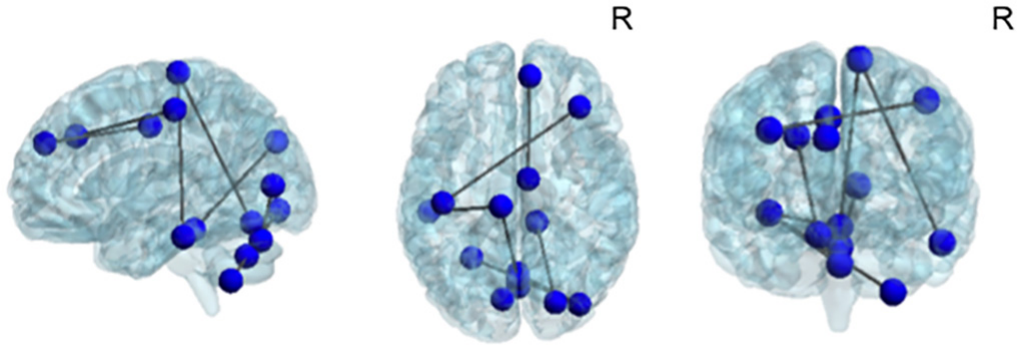
Graphical representation of the ToM-network. The network, composed by 14 nodes and 9 edges, was obtained by correlating whole-brain connectivity matrices with the ToM composite score across patients. Specifically, we found a significant positive correlation (p<0.05) between the nodes of the network and the ToM Composite score in DM1 patients. The brain network is visualized using the BrainNet Viewer (https://www.nitrc.org/projects/bnv/),[45]. See text for further details. Abbreviations: R = Right.

NBS analysis showed a significant positive correlation in DM1 patients between inter-nodal connectivity and ToMCs forming a network with 14 nodes and 9 edges (Table 4 and Fig 1). This network encompassed frontal (BAs: 6, 9/10; 23; 46), temporal (BA: 20), occipital (BAs: 17; 19) and cerebellar (Lobules 3, 8 and Vermis) areas. According to the Methods section, using the graph theory approach, this ToM-network was used as an atlas to investigate, the potential differences in the topological properties of DM1 brains compared to healthy brains. When comparing global topological properties of the ToM-network, no differences were found between DM1 patients and healthy controls. Conversely, when looking at local properties, DM1 patients compared to controls showed a significant increase of nodal efficiency and degree in the left inferior temporal gyrus (BA20).

**Table 4:** Network-based analysis: Difference of functional connectivity into pairwise brain regions in patients with Myotonic dystrophy type-1.

Pairwise brain regions	t-values*#
R BA9/10 ↔ R BA23	4.35
R BA46 ↔ L BA3	2.99
L BA6 ↔ L BA20	3.33
R BA19↔ R Cerebellum_lobule 3	4.08
R BA19 ↔ L Cerebellum_lobule 8	3.60
R BA19 ↔ Vermis 6	2.74
L BA6 ↔ Vermis 6	3.42
L BA17 ↔ Vermis 7	3.43
R BA19 ↔ Vermis 8	3.23

*T-values are reported.

^#^p-value <0.05 in the whole-network comparison using Network-Based Statistics [28];

Abbreviations: R = right; L = left; ↔ = bidirectional connections.

### Post-hoc analysis of BA20 connections

**Fig 2:**
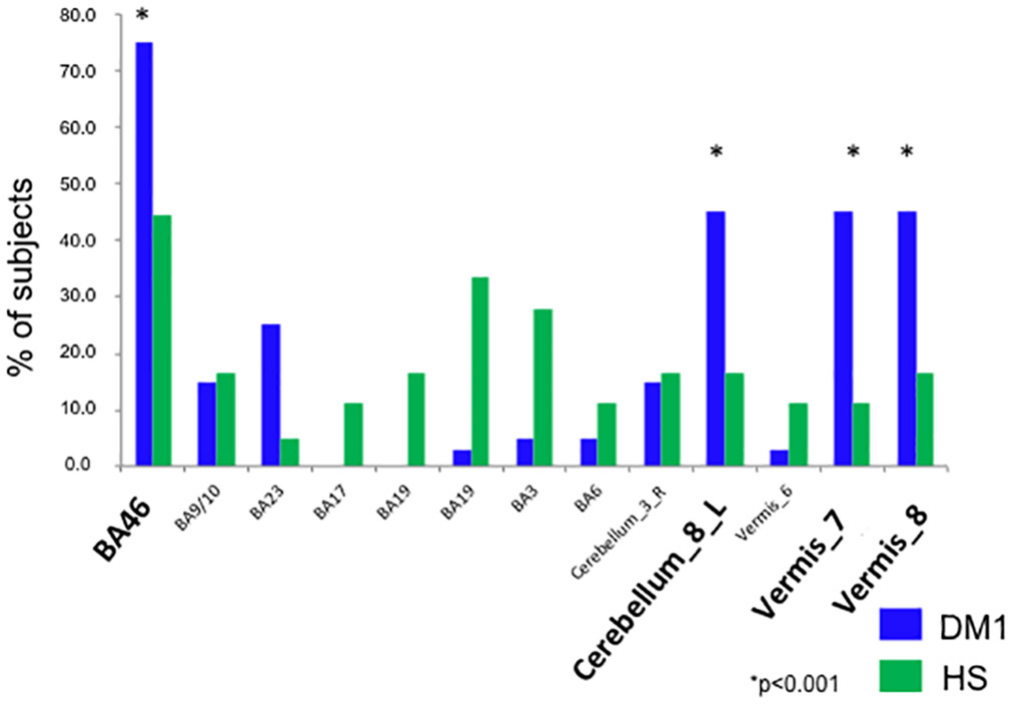
Frequency of occurrence of direct connections between BA20 and the rest of ToM network. The number of subjects (expressed as a percentage) showing a direct significant correlation between BA20 and one of the remaining 13 nodes involved in the ToM-network, is plotted for DM1 patients (in blue) and healthy controls (in green). Significant (p<0.001) differences between patients and controls, highlighted by stars, were observed in the connections between BA20 and BA46 and the cerebellum. Bold characters are used to highlight the nodes that show the strongest connectivity with BA20 in DM1 patients. Abbreviations: DM1 = Myotonic dystrophy type 1; HS = Healthy Controls; BA = Brodmann areas; * = Chi-square. See text for further details.

Considering this significant increase of centrality observed in patients’ BA20 only, we performed further analyses to better define the connectivity between this node and the remaining 13 nodes of the ToM network. Specifically, we computed for each participant the correlation between the mean temporal series from BA20 and that from each of the remaining 13 nodes. Positive correlations were classified as evidence of connection. For each of these connections, we counted the frequency of occurrence (i.e., how many subjects, in either patient or control group, exhibited a connection between BA20 and any of the 13 remaining nodes), and we compared those frequencies between groups using Chi-square. This analysis showed a set of significant differences between DM1 patients and healthy controls (Fig 2). In detail, BA20 resulted connected with BA46 in 75.0% of DM1 patients (15 out of 20) and in 44.4% (8 out of 18) of healthy controls (Chi-square = 8.18 d.f. = 1, p = 0.002); BA20 was connected with the cerebellum (lobule 8, vermis portions 6 and 7) in 45.0% (9 out of 20) of patients and in 11.1% (2 out of 18) of controls (Chi-square = 5,29, d.f. = 1; p<0.02). Conversely, connections between BA20 and occipital regions were observed in healthy subjects but in none of the patients with DM1.

## Discussion

Here we provide the first evidence of the existence of a brain network correlated with ToM dysfunction in patients with DM1. This fits with previous evidence of widespread structural and functional brain alterations in DM1 patients, which are dominated by white matter involvement [5, 9–10].

All our patients had an adult or childhood disease onset, and showed a mild to moderate muscular impairment, with no intellectual impairment as measured by WAIS-R. Including DM1 patients with a I.Q. level within the range of normality, allows to control for the impact of global cognitive efficiency on their performance at ToM tests. On the other hand, this limits representativeness of our sample to the entire DM1 population.

When assessing ToM, we observed a partial dissociation in patients’ performances, with a more remarkable impairment at ToM-story than RMET tasks. Specifically, most of the DM1 patients failed about one third of the available stories, while only a few of them failed at a greater or lesser extent. It is plausible to hypothesize that a greater number of errors is indicative of a more severe ToM impairment. Therefore, we speculated that in the present case DM1 patients covered a large spectrum of ToM impairment, showing up in most cases a moderate level of ToM dysfunction.

These two tests differ from each other for the different load of emotional and cognitive processing required. RMET is indeed based upon the emotional perception [29] of other people’s feelings as expressed by gaze, and characterizes the most affective aspect of ToM [30–31]. Conversely, the ToM-story task is based on the ability to infer and predict intentions, thoughts, desires, intuitions, behavioural reactions, plans and beliefs of other people [32], thus characterizing the highest executive functions underlying the ToM [30–31]. Many Authors have proposed that different mechanisms are needed to process emotional and cognitive aspects of ToM, hypothesising both sequential models, for instance the “mindreading system” of Baron-Cohen [33] and discrete models [34]. According to Baron-Cohen [35–36] the mentalizing abilities in human beings are developed sequentially, starting from the age of 18 months and reaching their full development in pre-adolescence [31]. According to this theory, most of our patients had an adult clinical onset, which might explain why their basic emotion perception process (developed before evident clinical manifestations) was less impaired, although it is possible that some of our patients may have encountered (undetected) daily living difficulties before the clinical onset of the disease. Overall, our neuropsychological findings confirm the only previous study based on ToM in DM1 patients [11], thus reinforcing the idea of a strong impact played by abnormal social cognition in the everyday life of these patients. Additionally, we found a significant correlation between patients’ RMET performances and their CTG triplets expansion. This suggests that genetics drives not only muscular and cognitive impairments in DM1 patients [1,5], but also their mentalizing abilities. Moreover, we observed a ceiling effect in both measures assessing ToM in the HS group. In our opinion this result reinforced the hypothesis that deficits in mentalizing observed in DM1 patients are related to the genetic pathology. However, the lack of ToM assessment in the whole control group is a limitation of this study that future work should address. Another limitation is intrinsic to the Italian version of ToM-story tests, which do not include control stories. This does not allow a direct distinction between comprehension and ToM deficits.

We identified here, for the first time in patients with DM1, a specific functional network sub-serving social cognition abilities. Within such a network, we identified in DM1 patients but not in healthy controls, a single node that is over-connected with a set of other brain areas previously regarded as critical for social cognition abilities (see [13]). An increase of connectivity in DM1 brains is not surprising, but it is consistent with previous findings obtained in an independent group of DM1 patients [4] or in patients with psychiatric disorders [37]. This abnormality can be interpreted as either a specific neurobiological substrate of the disease or a compensatory rearrangement.

The ToM-network we identified in DM1 patients involved several prefrontal (BAs 46, 9/10, 23), temporal (BA20), parietal (BAs 6, 3), occipital (BAs17, 19) and cerebellar (lobules 3, 8 and vermis) regions. All these areas have been previously reported to be implicated in ToM in fMRI and PET investigations [13,38]. Indeed, fMRI studies revealed that ToM abilities are sub-served by several brain structures, including the medial pre-frontal cortex, the orbito-frontal cortex, the cingulum, the temporal pole, the superior temporal gyrus, the inferior temporal gyrus, the temporo-parietal junction and the amygdala [13,38]. Against this background, our data suggest that ToM functions depend on the integrity of the whole network, and not of specific areas. Within this model, abnormal connectivity between more “central” nodes, or “hubs” is believed to cause more remarkable deficits than that between peripheral nodes. We therefore assessed the topological properties of the ToM-network in DM1 patients, and compared them with those measured in healthy controls. This analysis highlighted the “high centrality” of the inferior temporal gyrus (BA20), including the fusiform gyrus, in the information processing within the “ToM network”. Mar [13], performing an extensive meta-analysis on the neural basis of ToM, reported BA20 as activated in both story- and non-story-based tasks. Consistently, we found a prominent role played by BA20 as result of a correlation between brain functional connectivity and ToMCs, a composite score obtained by combining together RMET and ToM-story. When comparing the role of “hub” played by BA20 between DM1 patients and controls, more patients than controls showed connections between BA20 and prefrontal (BA46) and cerebellar regions. The dorsolateral prefrontal cortex (BA46) was found to be involved in ToM abilities in both lesion [39] and FDG-PET [40] studies. These studies highlighted the role of BA46’in cognitive aspects of ToM [40]. The cerebellum is also implicated in ToM [13,41]. In particular, the posterior cerebellar regions and vermis [42] have been shown to be related with ToM in both healthy controls [13] and patients with schizophrenia [41] and autism spectrum disorder [43].

When considering the global topological measures of the ToM-network, DM1 patients did not reveal significant differences with respect to controls. This is not surprising, as considerable brain disorganization must occur before global indices are affected [44]. Overall, our data reinforce the evidence of ToM deficits in patients with DM1. These dysfunctions affect day-to-day activities by compromising social interactions and personal relationships. We propose that, as well as for unusual personality traits [4], social dysfunction in DM1 patients may be a consequence of brain network abnormalities.

In conclusion, the current study describes, for the first time, the neural basis of ToM and its abnormalities in patients with DM1. This is of clinical interest considering that impaired abilities in social cognition may account for the most impacting disabilities of DM1 patients. Future investigations, based on current findings, should explore the potential of therapeutic interventions, such as cognitive training targeting mentalizing abilities, psychotherapy, and modulation of brain connectivity based on neurophysiological tools, such as transcranial magnetic stimulation or biofeedback. Early interventions might modify the future of DM1 patients with a substantial reduction of social costs, in the absence or in addition to disease-modifying therapies.

## Supporting Information

S1 File Supporting information table shows the individual composite ToM scores derived from principal component analysis in patients with DM1. (DOCX)

S2 File Adjacency matrices. Adjacency matrices obtained by graph theory bases analysis illustrating the intra-nodal correlations in individual subjects (DM1 patients and controls)
(ZIP)
