# Supplementary material for: “I Know that You Know that I Know”: Neural Substrates Associated with Social Cognition Deficits in DM1 Patients
Source: PLoS One. 2016 Jun 3;11(6):e0156901. doi: 10.1371/journal.pone.0156901 (PMC4892543; doi:10.1371/journal.pone.0156901)
Supplement: S1 File — (DOCX) [file pone.0156901.s001.docx]

**Supplementary table. Individual factorial scores derived from ToM tests obtained by DM1 patients**

| DM1 patients | ToM composite score |
| --- | --- |
| Subj01 | -1.95396 |
| Subj02 | -0.82260 |
| Subj03 | 1.07923 |
| Subj04 | 0.44246 |
| Subj05 | 2.39324 |
| Subj06 | -1.43807 |
| Subj07 | -0.01004 |
| Subj08 | 0.84610 |
| Subj09 | -0.11906 |
| Subj10 | 0.18994 |
| Subj11 | -0.31130 |
| Subj12 | 0.62443 |
| Subj13 | -0.43448 |
| Subj14 | -0.29164 |
| Subj15 | -0.31316 |
| Subj16 | -0.53938 |
| Subj17 | 0.65828 |
| Subj18 | -0.43448 |
| Subj19 | 0.62443 |
| Subj20 | -0.43448 |

DM1= Myotonic dystrophy type-1; ToM=Theory of mind.
